# Supplementary material for: Causal interplay between lactose intolerance and gut microbiota: a combined bidirectional Mendelian randomization and in vivo validation study
Source: Front Nutr. 2026 Jun 1;13:1803337. doi: 10.3389/fnut.2026.1803337 (PMC13265576; doi:10.3389/fnut.2026.1803337)
Supplement: Supplementary file 1 [file Data_Sheet_1.zip › supplementary materials/Forward/leave one out cross validation/ebi-a-GCST90027592.finngen_R12_E4_LACTONAS.pdf]

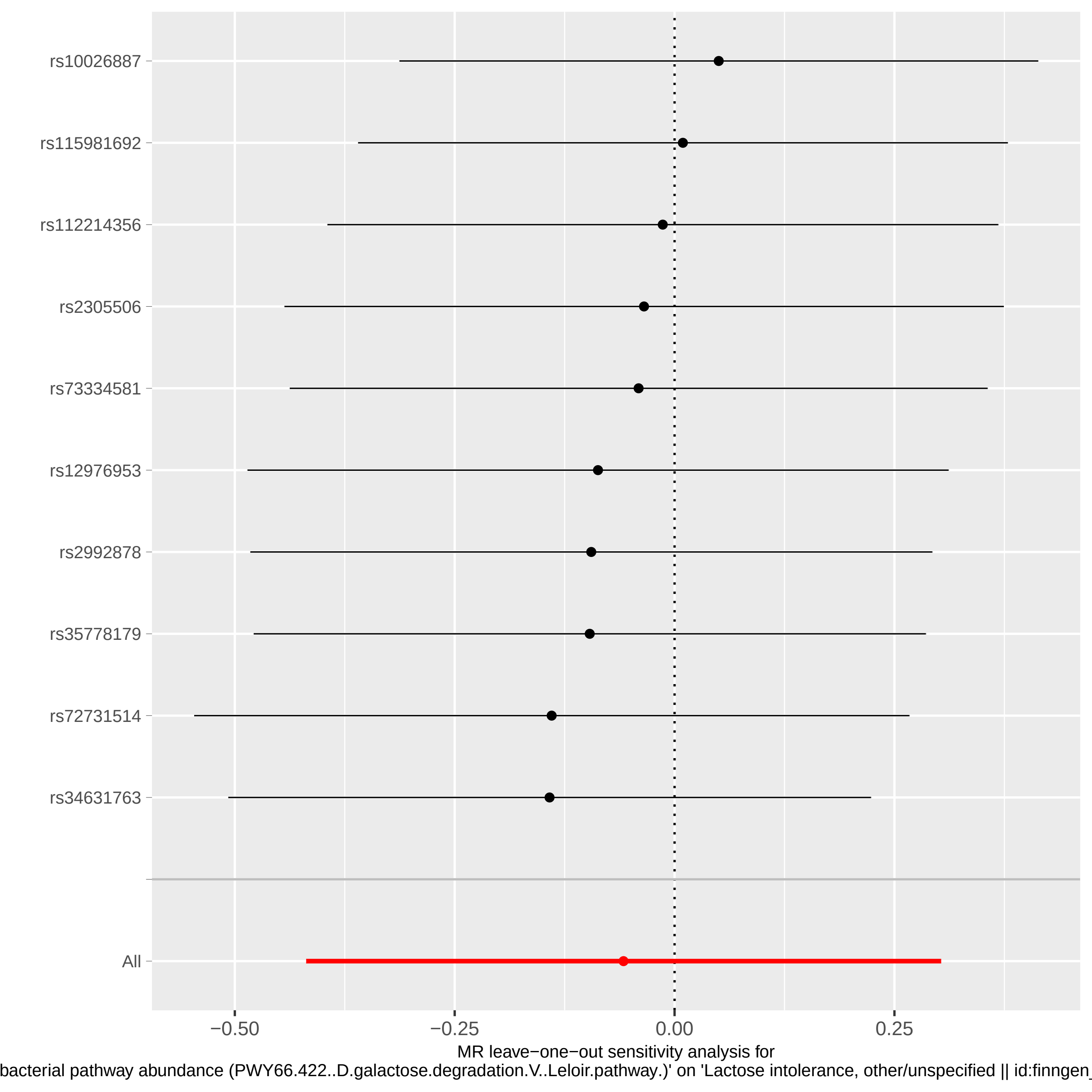

rs10026887
rs115981692
rs112214356
rs2305506
rs73334581
rs12976953
rs2992878
rs35778179
rs72731514
rs34631763
All
−0.50
−0.25
0.00
0.25
MR leave−one−out sensitivity analysis for
bacterial pathway abundance (PWY66.422..D.galactose.degradation.V..Leloir.pathway.)' on 'Lactose intolerance, other/unspecified || id:finngen_
